# Impact of the Trade-Related Aspect of Intellectual Property Rights Agreement on Pharmaceutical Industry in Developing Countries: A Scoping Review

**DOI:** 10.22037/ijpr.2021.115264.15282

**Published:** 2021

**Authors:** Mahdieh Fathi, Farzad Peiravian, Nazila Yousefi

**Affiliations:** *Department of Pharmacoeconomics and Pharma Management, School of Pharmacy, Shahid Beheshti University of Medical Sciences, Tehran, Iran*

**Keywords:** Pharmaceutical companies, liberalization rules, patent regime, Strategies, TRIPS

## Abstract

Pharmaceutical companies in developing countries are heavily influenced by the Trade-Related Aspects of Intellectual Property Rights (TRIPS) agreement and economic liberalization rules. To adjust to the new patent regime, pharmaceutical companies had to adopt some strategies. A systematic review was conducted on the experiences of the pharmaceutical industry in developing countries and strategies adopted by local pharmaceutical companies to survive after the TRIPS agreement. Scopus, PubMed, and ProQuest databases were searched, and twenty-five papers were reviewed. The pharmaceutical industry experiences have been classified into successful and unsuccessful experiences based on criteria developed by the authors. Firm strategies were also divided into four categories based on external and internal factors: aggressive, conservative, competitive, and defensive strategies. Companies were able to survive and even grow after the TRIPS agreement by rebuilding their structures, improving their competencies, and adopting appropriate strategies in line with the new conditions.

## Introduction

The impact of the healthcare industry on the economic growth of countries is remarkable. The economic development of a country relies on the health of society. Healthier people are more productive and more able to create opportunities to participate in economic growth (1). Any company or organization that provides clinical services, manufacture drugs, and medical equipment, and healthcare-related support services such as medical insurance are considered as the healthcare industry. The pharmaceutical industry, as the most important industry related to the healthcare industry, creates jobs for many people and contributes to the export earnings. As a result, a well-functioning pharmaceutical industry would lead directly to social wellbeing (2). This industry is recognized by UN Millennium Development Goals “as an actor that can contribute to economic development” (3). The pharmaceutical industry is one of the most profitable business sectors in the world which contributes as much as 7 to 10 percent to the development of the Gross Domestic Product (GDP) of the top 10 economies of the globe (2). Several papers have studied the impact of the pharmaceutical industry on the economic development of Low and Middle-Income Countries (LMICs) (4–7). In general, the production level of the pharmaceutical industry in the GDP of developing countries is 0.5% on average, which is not significant (7). The contribution of the pharmaceutical sector in India’s GDP is 2% and 12% of the manufacturing sector GDP by 2013 (3). India, as a developing country, has a large and developed pharmaceutical industry. Indian pharmaceutical industry accounts for an estimated 10% of global production. India ranks third in terms of volume and tenth in terms of value in the pharmaceutical sector of the global market (8).

Medicines are exceptionally high-tech commodities, most of which are directly life-related products. For these reasons, most governments or states control all aspects of medicines from their first trials in animals, through manufacturing and distribution to post-marketing pharmacovigilance.

The distinctive feature of this industry is that pharmaceutical products are so unique and irreplaceable by other goods (3) that the issue of Intellectual Property Rights (IPR) in this industry is particularly prominent. In the pharmaceutical industry, trade has been strongly linked to IPR to prevent imitation and to increase returns on research and development (9). 

With the establishment of the World Trade Organization (WTO) and the introduction of the Trade-Related Aspects of Intellectual Property Rights (TRIPS) agreement on January 1, 1995, and its transitional periods for developing countries that lasted till 2005, all member countries except the Least Developed Countries (LDCs) were required to adopt minimum standards of intellectual property (10–13). The expiration of the transitional periods for the LDCs to introduce the pharmaceutical patent was January 1, 2016, which was later extended until January 1, 2033 based on requests from the LDCs (15). Members have to make patents available for inventions, whether products or processes, based on the standard patent criteria (novelty, inventiveness, and industrial applicability) in all fields of technology, without discrimination between domestic and multinational industries (16).

Based on the Doha Declaration on the TRIPS Agreement and Public Health, the proper interpretation and implementation of the TRIPS Agreement would protect public health and promote access to medicines for all (16).

Economic liberalization and the TRIPS agreement are major challenges for pharmaceutical companies in developing countries. Due to ease of access to the global markets, there will be opportunities for sectors and companies that do not have much government support and have the required capabilities to compete with multinational companies. One of the main concerns for the world’s leading pharmaceutical companies is the different responses to intellectual property rights in different countries. The WTO agreement on TRIPS established uniform IP standards between countries to reduce the variability of IPR protection among countries (18).

Pharmaceutical regulations need to be reformed by WTO members to protect local generic producers. Some policy options should be adopted to ensure access to affordable medicines and save the local generic industries (15). Any wrong selection of rules or policies can lead to the destruction of many local pharmaceutical companies (19); instead, implementing appropriate policies will lead to the growth of this industry. Pharmaceutical companies should adapt to these policies to develop and grow by converting threats into opportunities or even by taking advantage of existing opportunities (20, 21).

The unique position of the Indian pharmaceutical industry among the countries in the developing world is due to its strong generic pharmaceutical industry, which produces medicines with the lowest price among the world. Since multinational corporations are experiencing slow growth in the production of new drugs and a large number of patented drugs are expected to be off-patent in the next few years, more attention has been paid to generic drugs (22). 

As a signatory member of the TRIPS agreement, India has experienced radical shifts in its pharmaceutical industry (23), but this agreement accelerates the Indian pharmaceutical industry’s movement towards innovative Research and Development (R&D) (16). A period of 10 years has been given to the Indian pharmaceutical industry to amend patent laws. A vast majority of Indian pharmaceutical firms have strengthened their positions in the domestic markets and international markets (24). Harmonizing pharmaceutical rules with the TRIPS agreement, using the flexibilities of TRIPS, and protecting national companies from unfair international policies and rules by the Indian government have led to suitable conditions for the growth and development of the Indian pharmaceutical industry. Hence, in the new patent regime, Indian companies have adopted different strategies, such as producing generic drugs, investing in research and development departments to create new medicines, partnership with multinational companies in research areas, marketing their patented drugs, and producing them through signing contracts with their owners (19). 

To prevent the domination of foreign pharmaceutical companies in the Chinese pharmaceutical market, the government has encouraged Mergers and Acquisitions (M&A) in the pharmaceutical industry to raise resources and form larger companies and has supported research and development projects through government subsidies (25). Investors in East Asia use China as an export platform for the western markets because China encourages foreign direct investment and Joint Ventures (JVs) to establish a commercial presence (9). The direct import content of exports by FJV’s in China is high (25), and trade links between China and the East Asian economies have been well strengthened due to the ownership structure of these enterprises and the high import content of their manufacturing (9).

Unlike India and China, the situation in the Brazilian pharmaceutical industry has not been particularly promising after the TRIPS agreement. Inaccurate enforcement of intellectual property laws may be one of the causes for the lack of innovation in the Brazilian pharmaceutical sector. On the other hand, early adherence to the TRIPS agreement has failed to encourage the emergence of technological innovations in the Brazilian pharmaceutical industry (27). This is due to the lack of infrastructure, rules, and conditions required for the new patent regime. It should be noted that in addition to the negative effects of the TRIPS agreement on the Brazilian pharmaceutical industry, the new IP regime has had some positive impact, which is the creation of new regulatory frameworks such as industrial property law and its revision, generic law, the establishment of the National Agency for Health Monitoring (ANVISA), and Innovation law (28). 

The Jordanian pharmaceutical industry has also experienced severe conditions after the accession to the WTO in 2000. This country was required to introduce TRIPS-plus provisions in its national patent laws after accession to the WTO in 2000. The US-Jordan Foreign Trade Agreement (FTA) is a new framework of TRIPS-plus rules which allows multinational pharmaceutical companies to prevent generic competition with their products. Data exclusivity, as a TRIPS-plus rule, prohibits generic competition for many medicines. The US-Jordan FTA was formally enacted on December 17, 2001 (12).

**New contribution**

Despite numerous studies about the impact of the TRIPS agreement on the pharmaceutical companies in developing countries, there has been no systematic evaluation of the experiences of the pharmaceutical industry in developing countries after the TRIPS agreement yet. The present study attempts to look at the successful and unsuccessful experiences of pharmaceutical industries in developing countries after the TRIPS agreement came into effect. In our review, we were particularly interested in studying strategies of local pharmaceutical companies in developing countries after the TRIPS agreement. This review seeks to address the strategies adopted by local pharmaceutical companies in developing countries to survive and thrive under the new patent regime.

## Methods

A scoping review is a type of study which has great potential to generate new knowledge by identifying, selecting, and analyzing secondary data. The five-step methodology proposed by Arksey and O’Malley (2005) has been used in this study. The steps include identifying the research questions, determining the research strategy, finding the relevant studies through electronic databases, selecting studies related to the issues, analyzing the data, and finally collating, summarizing, and reporting the results (29).

For the first step, we clearly defined the research questions:

What are the experiences of the pharmaceutical industry in developing countries after the TRIPS agreement?

What strategies are adopted by local pharmaceutical companies in developing countries to survive and thrive under the new patent regime?

As the second step, we went through the Scopus, PubMed, and ProQuest databases. Keywords selected in the search strategy included “world trade organization”, “WTO”, “Trade-Related Aspects of Intellectual Property Rights”, or “TRIPS”, and “pharmaceutical companies”, “pharmaceutical firms”, or “pharmaceutical industry”. This stage was carried out in December 2019; nevertheless, there was no time limitation in selecting articles.

**Figure 1:**
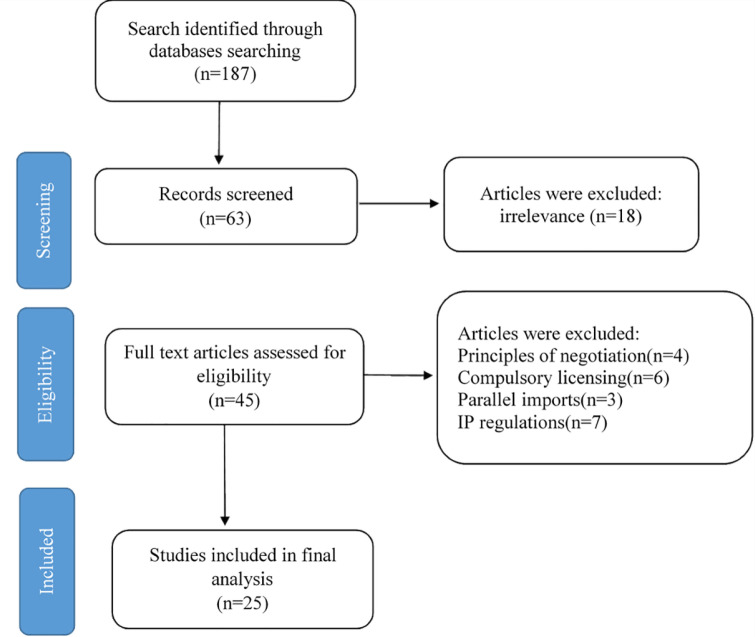
The screening and selecting articles in accordance with the PRISMA statement

In the third step, the initial search was conducted, and after eliminating the duplications, 187 studies were collected. We tried to select the most related articles which met our criteria. Therefore, we have restricted our search to the papers which include “developing countries” in English. After the exclusion, we were left with 63 papers. To reinforce the validity of the findings and decrease subjective bias, two members of the research team reviewed and assessed the titles and abstracts of included documents independently. In case of disagreement, the research members first tried to convince each other, and if they did not reach a consensus, the third person from the research team would make the decision as to the suitability of the article for inclusion. In this stage, papers regarding the accessibility of drugs and the rules of trade in the post TRIPS era were excluded since they did not meet the criteria. Therefore, 45 studies were selected. Eventually, after reading the full manuscripts of the remaining studies, unrelated papers were eliminated, and 25 studies were selected for the final analysis. Irrelevant papers included 4 articles about the principles of negotiation with multinational pharmaceutical companies, 6 articles concerning rules of compulsory licensing,3 articles about parallel imports, 7 articles regarding existing laws and regulations about data protection and IP in developing countries. Figure 1 is the PRISMA statement of the process of the study selection.

In the following, we show what experiences the pharmaceutical industry has had in developing countries after the TRIPS agreement, and we have divided these experiences into two categories of successful and unsuccessful. Then, the firm strategies of countries with successful experiences are shown in the Strength, Weakness, Opportunity and Threat (SWOT) and Internal & External (IE) matrix.

SWOT analysis is an essential instrument for decision-making and is commonly used to systematically analyze an organization’s internal and external environments (29). This technique is attributed to Albert Humphrey, who led a convention at Stanford University in the 1960s and 1970s using data from Fortune 500 companies (31, 32).

SWOT analysis divides the main parts of the information into two major categories: (A) Internal factors: These factors indicate the strengths and weaknesses of the organization. Strengths and weaknesses are factors in the system that enable the organization to either achieve its goal or avoid it. (B) External factors: These are opportunities and threats imposed on the organization by the external environment. Opportunities and threats, as external factors, facilitate or limit the organization in obtaining its goals, respectively (33).

The SWOT analysis is used to understand the various conditions in the organization, to determine the strategies, and finally, to choose the best strategy (34).

SWOT Matrix Formation and Strategy definition: SO strategies consist of internal strengths with external opportunities. WO strategies consist of internal weaknesses with external opportunities. ST strategies consist of internal strengths with external threats. WT strategies consist of internal weaknesses with external threats.

Internal and External Analysis by Internal–External Matrix: IE matrix is used to analyze internal and external factors concurrently and to determine the strategic position of an organization (34). The proportional area of strategies in the SWOT framework is highlighted by the IE matrix.

## Results

**The experiences of the pharmaceutical industry**

After the TRIPS agreement, the experiences of the pharmaceutical industries in developing countries differ from one country to another. The industrial and technological policies and, consequently, the development strategies adopted by pharmaceutical companies are quite different, leading to different experiences in the pharmaceutical industry in developing countries.

The experiences of the pharmaceutical industry in developing countries after the TRIPS agreement is described in Table 1. 

The experiences of the pharmaceutical industry in developing countries have been classified into successful and unsuccessful experiences based on such criteria as the number of Drug Master File (DMF) and Abbreviated New Drug Application (ANDA), filing or licensing activities, R&D intensities, innovations, R&D expenditures, different types of alliances with Multinational Companies (MNCs), introduction of New Chemical Entities (NCEs), generic competition, number of Contract Research And Manufacturing Services (CRAMS), and government supportive policies. 

Table 2 contains the criteria explaining successful and unsuccessful experiences of the pharmaceutical industry in developing countries after the TRIPS agreement.

**Figure 2:**
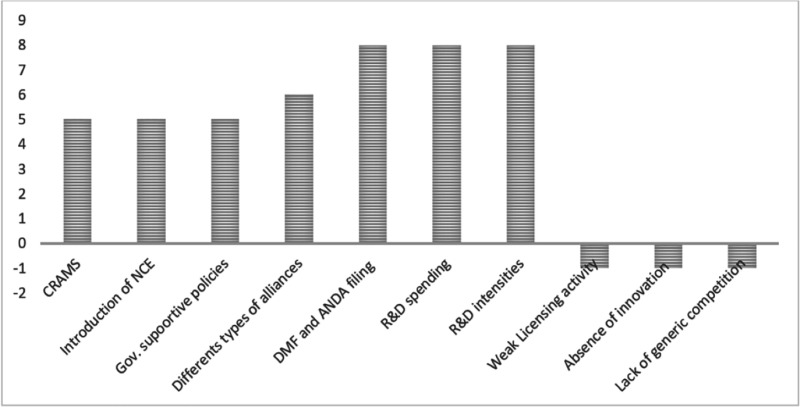
The successful and unsuccessful experiences of the pharmaceutical industry in developing countries after the TRIPS agreement. CRAMS= Contract Research and Manufacturing Services, NCE = New Chemical Entities, DMF= Drug Master Files, ANDA= Abbreviated New Drug Application, R&D = Research, and Development

Although the pharmaceutical industry experiences in developing countries are different after the TRIPS agreement, the frequency of each experience varies in the studied papers. The most frequent successful and unsuccessful experiences of the pharmaceutical industry in developing countries are demonstrated in Figure 2. Successful experiences are shown at the top of the chart with positive numbers, and unsuccessful experiences are shown at the bottom of the chart with negative numbers.

**The strategies adopted by pharmaceutical companies**

Every company is dealing with various conditions, which can comprise potential stimulants or possible obstacles to the company’s performance or the objectives the company wishes to achieve. Adopting strategies tailored to different requirements plays a vital role in the success of firms. The correct interaction of business management with its environment leads to successful performance in a company. This environment can be internal or external. The external environment consists of variables existing outside the company. Examples of these variables, which are considered as a direct environment, are the shareholders, the government, the suppliers, the local authorities, the competitors, and the clients. The examples of variables that are considered as indirect environments include economic, socio-cultural, technological, political, and juridical influences. The internal environment of the company consists of variables within the company itself, including the company structure, the company culture, and the resources of the company (52). 

**Figure 3:**
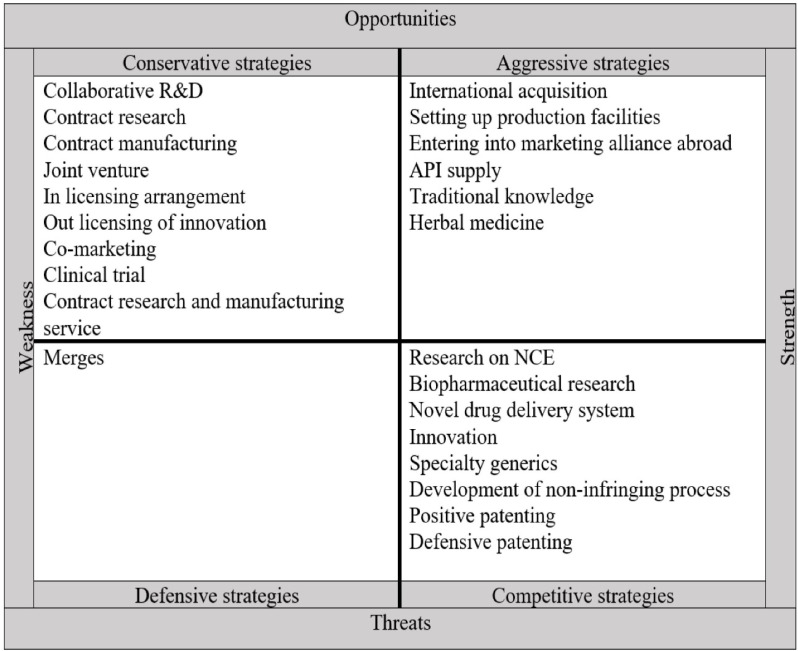
Internal–External (IE) matrix and strategies adopted by pharmaceutical companies after the TRIPS agreement. API = Active Product Ingredient, NCE = New Chemical Entities, R&D= Research, and Development

The strategies adopted by pharmaceutical companies after the TRIPS agreement are summarized and shown in Figure 3. Given the strengths and weaknesses of companies and the opportunities and threats that have arisen after the TRIPS agreement, firm strategies are divided into four categories based on the external and internal factors: aggressive, competitive, conservative, and defensive.

Aggressive strategies use internal strengths to take advantage of external opportunities. According to the IE matrix, aggressive strategies adopted by pharmaceutical companies after the TRIPS agreement consists of the international acquisition, setting up production facilities, entering into marketing alliance abroad, API supply, traditional knowledge, and herbal medicine.

Conservative strategies aim to exploit opportunities in the external environment to improve internal weaknesses. Conservative strategies of the pharmaceutical companies of developing countries after the TRIPS agreement based on IE matrix include collaborative R&D, contract research, contract manufacturing, joint venture, in-licensing arrangement, out-licensing of innovation, co-marketing, clinical trial, contract research, and manufacturing service.

Competitive strategies use the organization’s strengths to reduce the effects of external threats. According to the IE matrix, competitive strategies of pharmaceutical companies in developing countries after TRIPS agreement include research on NCE, biopharmaceutical research, novel drug delivery system, innovation, specialty generics, development of the non-infringing process, positive patenting, and defensive patenting.

The objective of the defensive strategies is to reduce internal weaknesses and avoid threats from the external environment. In this case, the organization tries to reduce its activities to maintain its survival, merge with other organizations, declare bankruptcy, or eventually dissolve.

## Discussion

Based on our preceding discussion, the pharmaceutical industries in developing countries have had various experiences in the post TRIPS agreement period. The pharmaceutical industries in some countries have had successful experiences and have continued to grow and survive after the TRIPS agreement. In contrast, the pharmaceutical industries in some countries have had unsuccessful experiences and have failed to grow in the new patent regime and market liberalization. 

For example, Brazil enacted the rules for liberalizing its economy in the 1990s and followed the new technology regime only two years after the TRIPS agreement. As White and Linden (2002) have stated, organizations would not survive if the pace of economic reforms exceeded the pace at which organizations can adopt appropriate strategies (53). These problems include (1) prohibiting the local production of patented drugs and replacing them with imported products from the companies that have the patents of these drugs, (2) allocating resources to imported products instead of investing these resources in national R&D and purchasing other drugs, (3) inability to export local products due to the unpreparedness and incompatibility of Brazilian ports and airports with export rules (54). 

 Chittoor et al. (2008) have shown in their study that the prominent features of India’s institutional environment include a combination of private and public sector enterprises, substantial presence of foreign firms, better quality institutions, and gradual acceleration of institutional changes, which has led to local firms that have relatively higher knowledge or experience about market economies (24).

The strategies adopted by companies with successful experiences were studied in this paper. The pharmaceutical industry is shaped by external forces, such as economic reform, health system reform, industrial policy regimes, and changing business ethics alongside internal forces, such as enterprise competition (25, 39). 

As mentioned earlier, choosing a proper strategy should be in line with the external and internal environment of firms. Due to their strengths and weaknesses, pharmaceutical companies of developing countries are taking advantage of the opportunities created in the new patent regime, penetrating new and regulated markets, and enhancing their capabilities and innovativeness, either by collaborating or by competing with MNCs.

Pharmaceutical companies that are strong enough in drug discovery research activities begin to compete with the world’s leading pharmaceutical companies in the new patent regime. Therefore, they follow competitive strategies such as research on NCE, biopharmaceutical research, novel drug delivery system, innovation, specialty generics, development of the non-infringing process, positive patenting, and defensive patenting.

The availability of financial resources is an essential constraint that companies face in competing with multinationals. Local firms’ strategies for building internal capabilities and meeting international standards include modernizing manufacturing plants, investing in R&D, and importing technology depending on the availability of financial resources (25).

Because of the limited funds, pharmaceutical companies in developing countries collaborate with MNCs through contract agreements to obtain foreign funds to build their own product-research cultures and competitive capabilities (55). Hence, strategies like collaborative R&D, joint venture, in-licensing arrangement, out-licensing of innovation, co-marketing, clinical trial, contract research and manufacturing service have been followed by these firms. On the other hand, many MNCs tend to cooperate with local pharmaceutical companies in developing countries to reduce costs, increase development capacity, and shorten the “market entry time” for new drugs.

In his study, Rai (2008) shows that the Indian pharmaceutical industry is adopting a mix of competitive and collaborative business and R&D strategies to overcome the challenge posed by the new patent regime. He mentions that business strategies, comprised of in-licensing, out-licensing, and co-marketing alliances, are adopted by firms that have branded products but do not have adequate sales networks. They would be able to use the existing sales network of alliance companies by entering into such alliances. He also mentioned that Indian companies are increasingly seeking to create in-licensing arrangements with MNCs to launch their products in India. The arrangement covers a wide range of relationships, from marketing relationships (including Joint Ventures) to local production by Indian companies and sharing part of the profits with MNC. To produce innovative products, the in-licensing arrangement strategy is cheaper and less risky than buying companies or conducting research and development, which are much more expensive. Companies would be able to bring novel drugs into the country at a reasonable price with the help of the in-licensing strategy. Since these products have already been approved for marketing in other countries, they will be directly studied in the bio-equivalence study and phase-III trial, so regulatory procedures are more accessible and faster.

He also believes that companies trying to increase their market share and expand into regulated markets are pursuing strategies such as international acquisition, creation of production facilities abroad, and marketing alliance abroad (40). Active Product Ingredient (API) supply, focus on traditional knowledge, and emphasis on herbal medicine are other aggressive strategies that pharmaceutical companies in developing countries have adopted to expand their market in the new patent regime. On the other hand, small pharmaceutical companies that cannot compete with multinationals have merged with MNCs in the form of a conservative strategy (22).

Rai (2008) has argued that pharmaceutical firms have adopted two sets of strategies: High-End Competitive Strategies (HECS) and Low-End Competitive Strategies (LECS). HECS focuses on innovation, research on NCE, positive patenting, defensive patenting, and biopharmaceutical research, while LECS emphasizes specialty generics, non- infringing processes, and Novel Drug Delivery Systems (NDDS). Compared to LECS, HECS requires higher investment and more technical knowledge and skills (40). 

Yin *et al.* (2003) have stated that among the strategies adopted by Chinese pharmaceutical companies, M&A had the most significant impact on the pharmaceutical industry and market. M&A led to the development of better management methods. Many failed pharmaceutical companies survived by being sold to major companies. Thus, the risk of bankruptcy and downsizing is avoided (25).

Globalization causes fundamental changes in the external and internal environment of enterprises. The reduction of trade restrictions and the inflow of foreign investment are the critical components of economic liberalization. Reducing trade tariffs leads to less protection of domestic companies against imports (24). New regulatory environments or radical innovations are changes that occur in fast-changing business environments. Various studies have testified that after the TRIPS agreement, the different strategies and policies for the pharmaceutical sector were adopted by companies in developing countries. Companies were able to survive and even grow in the face of remarkable changes resulting from the TRIPS agreement by rebuilding their structures, improving their competencies and capabilities, and adopting appropriate strategies in line with the new conditions. Comparing the pharmaceutical industry experiences in developing countries related to industrial development and technological capability after the TRIPS agreement indicates that adopting suitable strategies is not enough to succeed in the industry. Factors such as the industry background, government approach to industry development and technological capability, market liberalization, technological infrastructures, human resources, sectoral regulation, and macroeconomic aspects help companies survive and thrive under the new patent regime. The impact of government-adopted industrial policies to support the pharmaceutical industry following the TRIPS agreement is significant in developing countries. To increase R&D spending, the governments have provided incentives such as various tax exemptions for the pharmaceutical companies and have facilitated collaboration between the private sector and the publicly funded research institutes (10, 39).

**Table 1:** Different kinds of experiences in the pharmaceutical industry in developing countries after the TRIPS agreement

	**Author/s (Year)**	**Country**	**Experience**
Successful	Atul Gupta (2000)	India	Indian companies have filed more DMF and ANDA cases with the US FDA in the post-TRIPS period (36).
	Kensuke Kubo (2004)	India	R&D intensity and the patent to R&D ratio have increased after 1995 (36).
	Alka Chadha (2006)	India	To secure non-infringing process patents in foreign countries, maximum resources are being spent by Indian firms (38).
	Biswajit Dhar and K M Gopakumar (2006)	India	Indian pharmaceutical companies have grown after 1995. The consolidation of Indian firms has improved since the beginning of the current decade. R&D spending of some of the leading firms has increased, and consequently, R&D intensities of the firms have improved significantly (10).
	Dinar Kale and Steve Little (2007)	India	Duplicative imitation, creative imitation, and collaborative R&D made the Indian pharmaceutical industry to move from basic R&D capabilities to advanced level R&D capabilities (39).
	Rajnish Kumar Rai (2008)	India	The industry is pursuing a combination of a competitive and collaborative business and R&D strategies in the new business environment (40).
	Raveendra Chittoor et al (2008)	India	Indian pharmaceutical firms have used the internationalization of resources and product markets. The indigenous growth model has been followed by Indian pharmaceutical firms (24).
	M D Nair (2010)	India	India has the most number of FDA approved manufacturing plants, the most number of DMFs and ANDAs in the US, and three or four blockbuster drugs from indigenous or collaborative R&D (41).
	Madhur Mohit Mahajan (2011)	India	Firms have moved toward the development of advanced-level process and product R&D capabilities. Many Indian pharmaceutical companies have recognized the difference of knowledge-based, organizational practices in imitative and innovative R&D quickly. They have gone for suitable measures like investing more resources into product and process development (42).
	Sunita Mishra and Ravi Kiran (2012)	India	Better technology, increase in in-house R&D, higher R&D performance, increase in the proportion of turnover spent on R&D, and increase in the therapeutics of the drugs have occurred in Indian firms after the TRIPS agreement (43).
	Satyanarayana Rentala *et al.* (2014)	India	The Indian pharmaceutical industry is experiencing an increasing trend of export competitiveness after 2005 (23).
	Sunil K Sahu (2014)	India	Outsourcing, consolidations, mergers, acquisitions, CRAMS, and other kinds of alliances and tie-ins have risen significantly in the post-TRIPs era (22).
	Salla Sariola *et al. *(2015)	India	The increase in clinical trial activity has been more than the introduction of NCE after the TRIPS agreement (44).
	Teg Alam and Rupesh Rastogi (2016)	India	More spending on R&D activities and strengthening the core competencies have yielded improvement in the financial position of pharmaceutical companies in the post TRIPS period (16).
	Mark Duggan *et al.* (2016)	India	Significant increases in pharmaceutical prices or the dramatic consolidation of the market did not happen by product patents in the new patent regime (18).
	Ravi Kiran (2017)	India	Product innovation, process innovation, and R&D intensity are being increased slowly by small and medium-scale pharmaceutical firms after the TRIPS agreement. To enhance their competitiveness, firms continue to rely on government policies rather than organizational policies (45).
	Hongjun Yin J and Warren Salmon (2003)	China	The M&A phenomenon was very helpful in order to save many state-owned pharmaceutical companies and improve the performance of the entire pharmaceutical industry (25).
Unsuccessful	Maria Pluvia Zuniga and Emmanuel Combe (2002)	Mexico	Licensing activity in the Mexican pharmaceutical industry is insufficient because of the weak interest or the weak usage of patent data (46).
	Rohit Malpani (2009)	Jordan	Generic competition of medicines launched by multinational pharmaceutical companies has delayed due to the data exclusivity, which is a TRIPS-plus rule. Jordanian generic companies are not encouraged by TRIPS-plus regulations to participate in drug research and development (12).
	Daniel Benoliel and Bruno Salama (2010)	Brazil	One of the reasons for the absence of innovation in the Brazilian pharmaceutical industry is the lack of more strict enforcement of intellectual property laws as well as the early adherence to the TRIPS agreement (27).

**Table 2:** The criteria with the explanation of successful and unsuccessful experiences of the pharmaceutical industry in developing countries after the TRIPS agreement

	**Criteria **	**Explanation**
**Successful experiences**	DMF and ANDA filing	Increase in the number of DMF and ANDA filing with FDA to enter into regulated markets indicate the R&D capability and bulk drug export intensity of Pharmaceutical industry (47).
	R&D spending	Increase in R&D expenditures is expected to have positive and significant impact on export competitiveness (23).
	R&D intensities	R&D intensity is defined as the ratio of a firm’s R&D investment to its revenue (48).
	Different type of alliances	Entering into different types of alliances can create an international-level, innovation-based drug industry (44).
	Government supportive policies	The results of a research study (2007) by EXIM Bank’s Occasional Paper Series showed that favorable government policies along with industry/firm level initiative have helped the industry to grow over the years (49).
	Introduction of NCE	NCEs are the result of highly sophisticated research and demonstrate the most advanced capabilities (39).
	CRAMS	CRAMS is a new growth strategy for pharmaceutical companies (22) to provide additional sources of revenues, access to new technologies, marketing networks, and best business practices abroad (50).
**Unsuccessful experiences**	Weak licensing activity	Due to the weak R&D activity, the number of patenting of domestic firms is less than 1 % of total numbers after the TRIPS agreement (51).
	Absence of innovation	Intellectual property protection acts as a tool that fosters domestic innovation (27), so lack of innovation refers to inability to generate new products and process innovations (39).
	Lack of generic competition	Data exclusivity, which is a TRIPS-plus rule, has delayed generic competition of medicines launched by multinational pharmaceutical companies (12).

## Conclusion

This study aims to review the experiences of the pharmaceutical industry in developing countries after the enactment of the TRIPS Agreement. The paper focuses on different firm strategies adopted by successful local companies to respond to the new patent regime. Comparing the experiences of the pharmaceutical industry of developing countries, it can be concluded that the varying positions of the pharmaceutical industry of developing countries in the global market are due to the significant differences in the strategies adopted by the pharmaceutical companies in those countries. Considering internal and external factors, it can be concluded that firm strategies were divided into four categories: aggressive, competitive, conservative, and defensive. Each category included the most suitable strategies that are in line with the specific situation of the company and its environment.

## Funding

This research did not receive any specific grant from funding agencies in the public, commercial, or not-for-profit sectors.

## Competing interests

The authors have no competing interests to declare.

## Author’s contributions

Implementing the research strategy and methodology, searching through databases, analyzing data, and preparing the paper draft have been conducted by MF. NY gave consultation on the design of the study and has defined keywords boundaries, and reviewed and commented on the manuscript. FP has supervised the methodology. All authors read and approved the final manuscript.
